# Effects of platelet-rich fibrin and piezosurgery on impacted mandibular third molar surgery outcomes

**DOI:** 10.1186/s13005-015-0081-x

**Published:** 2015-07-26

**Authors:** Lokman Onur Uyanık, Kani Bilginaylar, İlker Etikan

**Affiliations:** Department of Oral and Maxillofacial Surgery, Near East University Faculty of Dentistry, Nicosia, Cyprus; Department of Biostatistics, Near East University Faculty of Medicine, Nicosia, Cyprus

**Keywords:** Platelet-rich fibrin, Piezosurgery, Impacted third molar surgery, Pain, Swelling, Trismus

## Abstract

**Introduction:**

The aim of this study was the comparision of postoperative outcomes in impacted mandibular third molars that were treated using either platelet-rich fibrin (PRF), a combination of PRF and piezosurgery, or conventional rotatory osteotomy.

**Patient and methods:**

The study included 20 patients; 40 extractions of impacted mandibular third molars were performed. Patients were divided into two main groups. In group A (n = 20), traditional surgery was performed on one side (Group 1, *n* = 10); traditional surgery was performed, and PRF was administered to the extracted socket on the other side of same patient (Group 2, *n* = 10). In group B (*n* = 20), on one side, piezosurgery was used for osteotomy, and PRF was administered (Group 3, n = 10); on the other side of same patient, traditional surgery was performed (Group 4, n = 10). Parameters assessed at baseline for each patient included pain, the number of analgesics taken, trismus, and cheek swelling. These variables were also assessed on postoperative days 1, 2, 3, and 7.

**Results:**

Statistical analysis revealed a significant reduction in postoperative pain (sum of 1^st^, 2^nd^, 3^rd^ and 7^th^ days) and trismus (on postoperative day 1) in group 2 (traditional surgery + PRF group), and in postoperative pain, the number of analgesics taken (sum of 1^st^, 2^nd^,3^rd^ and 7^th^ days) and trismus (on postoperative day 1) in group 3 (piezosurgery + PRF group) compared to groups 1 and 4 (traditional surgery groups), (*p* ≤ 0.05). However, swelling on postoperative days 1, 3, and 7 did not differ among the groups (*p* > 0.05). Only difference was on second day between groups 1–4 and 2–4 (*p* ≤ 0.05).

**Conclusions:**

The results of our study have shown that the use of PRF with traditional surgery and PRF combined with piezosurgery significantly reduced pain during the postoperative period. In addition, PRF in combination with piezosurgery significantly decreased the number of analgesics taken. Both operations also significantly decreased trismus 24 h after the surgery. As a result of this study, PRF and combination use of PRF and piezosurgery have positive effects in reducing postoperative outcomes after impacted third molar surgery.

## Introduction

In oral surgery, impacted third molar surgery is one of the most common operations performed by oral and maxillofacial surgeons [1]. After the removal of impacted third molars, at the early postoperative periods, patients generally complain of pain, trismus and swelling which are the complications of this procedure [2, 3]. These inflammatory complications still remain an important factor for patients and surgeons are responsible for developing a strategy to reduce the risk of complications and improve postoperative healing [4]. Many attempts have been made to reduce postoperative outcomes following third molar surgery, including platelet-rich plasma administration [5], cryotherapy [6], preoperative and postoperative antibiotics [7], osteotomy using high or low speed rotary instruments [8], wound draining [9], the use of different kinds of flaps [10], postoperative ice packs [11], corticosteroids [12], analgesics [13] and laser [14].

Piezoelectric surgery has been proposed as an alternative to rotatory drilling instruments in oral surgery involving osteotomies [15]. Piezoelectric ultrasound osteotomy devices are very efficient when used at complex surgical sites, including soft tissues, nerves, and blood vessels due to their ability to selectively cut, which is effective on mineralized structures [1, 16]. The advantage of ultrasonic instruments is that they reduce trauma to hard tissues thanks to their highly accurate and conservative cutting action, which means that the procedure improves healing process [17]. As noted by Sivolella, such instruments have been employed in a wide range of procedures and surgical interventions, including maxillary sinus elevation, bone harvesting, alveolar crest enlargement, implantology, periodontal and orthognathic maxillofacial surgery, dental exposure and extractions, as well as ear, nose and throat surgery for the removal of cysts and tumours [18].

Platelet-rich fibrin (PRF) is a second-generation immune and platelet concentrate. PRF accrues all blood sample components supporting healing and immunity on just one fibrin membrane [19]. Dr. Joseph Choukroun was the first to address the application of platelet-rich fibrin in oral and maxillofacial surgery. He used autogenous whole blood to create a PRF clot with the help of a centrifuge [20]. Growth factors (Platelet-Derived Growth Factor (PDGF-ββ),Transforming Growth Factor (TGF-β1), Vascular Endothelial Growth Factor (VEGF), Insulin-Like Growth Factor 1), leukocytic cells and their cytokines (interleukin 1β [IL-1β], IL-6, IL-4, tumor necrosis factor α) are enmeshed within the PRF fibrin matrix [17]. PRF has been used in bone augmentation, angiogenesis, wound healing, and periodontal healing with promising results [21].

The present study evaluated and compared the effects of PRF, PRF combined with piezoelectric surgery, and rotatory instruments on the postoperative period after surgical mandibular third molar extractions.

## Patients and methods

This study was performed at the Near East University Faculty of Dentistry in Nicosia, Cyprus between January 2014 and August 2014. A total of 20 patients (10 female, 10 male) between 19 and 31 years of age participated. There was no statistically difference between groups in mean age, in group A; 22.65 and in group B; 22.35 (*p* = 0.974, p > 0.05). Bilateral extractions were required for all patients. The selection criteria were as follows: (a) the presence of bilateral, symmetrically oriented, impacted lower third molars requiring extraction for prophylactic reasons; (b) the absence of systemic diseases; (c) no chronic opioid use; (d) age >18 years; (e) non-smoker and non-alcoholic; (f) not pregnant; and (g) no allergy to penicillin or other drugs used during the standardized postoperative therapy. Patients taking antibiotics for a current infection, or who had acute pericoronitis or severe periodontal disease at the time of the operation and if tooth needed sectioning during the surgery, were also excluded.

Per the abovementioned criteria, only patients with bilaterally and vertically impacted lower third molars were included. Moreover, we selected cases with similar surgical conditions with respect to the location of the tooth relative to the jaw, the depth of impaction, the relationship with the ramus and inferior alveolar nerve. All patients required an osteotomy. Extractions of mandibular third molars were of moderate difficulty (class I, level C), and were assessed according to the classification system of Pell and Gregory [22].

Patients were divided into two main groups. In group A (4 male, 6 female, *n* = 20), on one side of patient, traditional surgery using burs was performed to Group 1 (traditional surgery, n = 10). Traditional surgery was performed, and PRF was placed in the socket of the extracted tooth on the other side of same patient in Group 2 (traditional surgery + PRF, n = 10). In group B (6 male, 4 female, *n* = 20), on one side, piezosurgery was used for osteotomy and PRF was administered to Group 3 (piezosurgery + PRF, n = 10), and on the other side of same patient, traditional surgery using burs was performed in Group 4 (traditional surgery, n = 10). Only difference between groups A and B was that we used piezosurgery in group 3. A minimum of 21 days separated the two operations in each patient for the return of parameters to preop-baseline prior to commencing second operation. The selection of processes of which technique to use first on each patient were randomly selected. Gender (*χ*^*2*^ = 1.6, *p* = 0.659) and operation side distributions (*χ*^*2*^ = 0.301, *p* = 0.960) did not differ among groups (p > 0.05).

Parameters examined in each patient, including pain, number of analgesics taken, trismus, and cheek swelling, were evaluated at baseline (prior to surgery) and on postoperative days 1, 2, 3, and 7. All of the examinations were undertaken at approximately the same time of day and by the same surgeon.

All of the patients were informed regarding the surgical procedure, postoperative time, and possible complications. The protocol design was approved by the local ethics committee (project number, NEU/2014/19–101). All of the participants provided written informed consent.

### Preparation of PRF gel

PRF was prepared according to the technique described by Choukroun et al. [23]. Approximately 15 min before surgery, a blood sample was taken without anticoagulant in 10 mL glass-coated plastic tubes that were immediately centrifuged (Elektro-mag M415P) at 3,000 rpm for 10 min (approximately 400 g). The platelet-poor plasma that accumulated at the top of the tubes was discarded. PRF was dissected approximately 2 mm below its contact point with the red corpuscles situated beneath, to include any remaining platelets that may have localized below the junction between the PRF and red corpuscles [24]. For each patient, 10 ml tube produced one PRF clot which was adequate to fill one extracted socket.

### Surgical procedure

All of the patients underwent a radiological examination, including panoramic radiography, and all were treated by the same surgeon and assistant.

In both groups the flap incision was triangular in shape which avoids muscle involvement (Archer flap). Surgery was performed under local anesthesia, using nerve blocking agents in the inferior alveolar, lingual (regional anesthesia) and buccal nerves (infiltration anesthesia), which contained 0.012 mg/mL epinephrine HCl and 40 mg/mL articaine HCl (2 mL Ultracaine D-S Forte Ampul; Sanofi Aventis). All of the third molar extractions were performed by raising a full-thickness mucoperiosteal flap. The surgeon used an identical approach during both surgeries, changing only the instrument used. After mucoperiosteal flap reflection, in groups 1 and 4 (traditional surgery groups), osteotomy was performed using a 1.6 mm round bur mounted on a W&H implanted surgical high-speed handpiece, at 40,000 rpm under abundant irrigation. All parts of the tooth were loosened with a lever and then removed. In Group 2 (traditional surgery and PRF group), osteotomy was performed using a bur; after the tooth was removed, PRF was placed in the extracted socket. In group 3 (piezosurgery and PRF group), osteotomy was performed using a piezoelectric device (EMS-Piezon Master Surgery) with an SL 2 cutting insert. Following removal of the tooth, PRF was placed in the extracted socket.

In all of the cases, the postextraction residual cavity of the impacted third molar was cleaned with sterile physiologic saline solution containing no antibacterial agents; 3–0 silk sutures (4 stitches) were used for wound closure and sutures were removed after 7 days. A gauze pack was pressed against the surgical site for the patient to bite on for 30 min. An icepack was then applied to the surgical area for 6 h immediately following surgery, according to an alternating 15 min on-15 min off schedule. No pharmacologic therapies or antibiotics were administered prior to surgery. Postoperatively, the same postoperative instructions were given to all patients: soft and cold diet for 24 h and they were instructed to take amoxicillin (500 mg) three times per day for 5 days, and to use antiseptic (Povidone-Iodine %7.5) mouthwash three times per day for 7 days. Acetaminophen (500 mg) was prescribed postoperatively, to be taken as required (500 mg every 4–6 h).

### Evaluation procedure

Pain was assessed during the postoperative periods using visual analog scales (VAS) ranging from 0 (absence of pain) to 10 (most severe pain) in conjunction with a graphic rating scale [8]. The number of analgesic tablets consumed was also recorded. Trismus was evaluated by measuring the distance between the mesial incisal corners of the upper and lower right incisors during maximum mouth opening as described by Ustun et al. [25]. Swelling was recorded clinically using a modification of the tape measure method described by Gabka and Matsumara [25, 26]. Three preoperative measurements were obtained between the following five reference points: the tragus, soft tissue pogonion, lateral corner of the eye, angle of the mandible, and outer corner of the mouth. Measurements were obtained on postoperative days 1, 2, 3, and 7. The preoperative sum of the three measurements was considered the baseline for that side. The difference between each postoperative measurement and the baseline value indexed the facial swelling and trismus for that day [25]. The time of surgery was considered the period between onset of incision and termination of suturing. All patients were seen on each of the four postoperative days and measurements were always obtained by the same individual, both preoperatively and postoperatively, on days 1, 2, 3, and 7 at approximately the same time of day (these measurements were done for each operation, n = 40).

### Statistical analysis

The Chi-square test was used to compare qualitative variables. To compare differences between more than two independent variables, One-Way ANOVA was employed. If group differences were statistically significant, they were compared bilaterally using Least Significance Difference (LSD) test. If the data were not normally distributed, the Kruskal-Wallis test was used instead of One-Way ANOVA. If group differences were statistically significant, the Mann–Whitney *U* test was used bilaterally, with the Bonferroni correction also applied. A value of *p* ≤ 0.05 was taken to indicate statistical significance. Statistical analyses were performed using the R statistical software package (ver. 2.14.0).

## Results

All of the patients tolerated the medication well, with no serious complications or side effects. Wound healing was uneventful in all patients. The mean times (minutes) it took for extractions were: 21.42 (traditional surgery = group 1), 21.82 (traditional surgery + PRF = group 2), 29.99 (Piezo + PRF = group 3), 24.24 (traditional surgery = group 4). There was no significant group difference in surgery duration (*F* = 1.249, *p* = 0.306) (p < 0.05).

### Pain

There was a significant difference between the VAS pain scores (added across 7 days) of groups 1 and 2 (*p* = 0.001) and groups 1 and 3 (*p* = 0.0001), but not between the scores of groups 1 and 4, (*p* = 0.762) or groups 2 and 3 (*p* = 0.791). Significant VAS differences in pain scores were also seen between groups 2 and 4 (*p* = 0.008), and groups 3 and 4 (*p* = 0.017). The mean value of VAS scores was 74.60, 25.00, 24.45, 48.51 from group 1–4, respectively (Table 1).

**Table 1:** Group comparison of VAS pain scores (added across 7 days) (mm)

Groups (G)	VAS (*n*)	Min.	Max.	Median	Mean	SD	*χ* ^*2*^	p*	U**	p*
Traditional (G1)	10	37.0	142.0	68.50	74.60	35.21			6.0 (G1–G2)	0.001*
Traditional + PRF (G2)	10	6.0	59.5	20.50	25.00	18.99	18.563	0.001	3.0 (G1–G3)	0.0001*
Piezo + PRF (G3)	10	0.0	43.50	28.5	24.45	14.95			46.0 (G1–G4)	0.762
Traditional (G4)	10	12.0	149.5	66.75	48.51	44.15			46.5 (G2–G3)	0.791
									15.0 (G2–G4)	0.008*
									18.5 (G3–G4)	0.017*

Abbreviations: *VAS* visual analog scale, *Min* minimum, *Max* maximum, *SD* standart deviation, *χ* ^*2*^ Kruskal Wallis test result; *p ≤ 0.05, significance **Mann–Whitney *U*-test (Bonferroni-correction) results between groups

There was a significant difference between the total number of analgesic doses taken (added across 7 days) by groups 1 and 3, (*p* = 0.015), and groups 3 and 4, (*p* = 0.033). No differences were observed among any of the other groups (*p* > 0.05). The mean value of the number total analgesics doses taken was 9.4, 5.6, 4.3, 9.5 from group 1–4, respectively (Table 2).

**Table 2:** Total number of analgesic doses in 7 days for groups

Groups (G)	Anal. (*n*)	Min.	Max.	Median	Mean	SD	*χ* ^*2*^	p*	U**	p*
Traditional (G1)	10	3.0	16.0	10.0	9.4	4.81			27.0 (G1–G2)	0.079
Traditional + PRF (G2)	10	1.0	10.0	5.5	5.6	3.02	8.436	0.038	18.0 (G1–G3)	0.015*
Piezo + PRF (G3)	10	1.0	10.0	3.5	4.3	2.94			49.5 (G1–G4)	0.969
Traditional (G4)	10	3.0	20.0	11.0	9.5	6.11			36.0 (G2–G3)	0.268
									32.0 (G2–G4)	0.167
									22.0 (G3–G4)	0.033*

Abbreviations: *Anal* Analgesics, *Min* minimum, *Max* maximum, *SD* standart deviation, *χ* ^*2*^ Kruskal Wallis test result; *p ≤ 0.05, significance **Mann–Whitney *U*-test (Bonferroni-correction) results between groups

### Trismus

There was a significant difference in the extent of trismus between groups 1 (% 25.61) and 2 (%9.03), (*p* = 0.011), groups 1 (% 25.61) and 3 (%9.3), (*p* = 0.019), groups 2 (%9.03) and 4 (%26.16), (p = 0.019) and groups 3 (%9.3) and 4 (%26.16), (*p* = 0.043) 24 h after surgery (Table 3). There were no statistically significant differences between the groups on any other days (*p* > 0.05; Table 4).

**Table 3:** Average of postoperative trismus after 1 day of surgery (%)

Groups (G)	Trismus (*n*)	Min.	Max.	Median	Mean	SD	*χ* ^*2*^	p*	U**	p*
Traditional (G1)	10	8	59	23.80	25.61	16.65			17.0 (G1–G2)	0.011*
Traditional + PRF (G2)	10	0	38	4.50	9.03	12.50	10.88	0.012	19.0 (G1–G3)	0.019*
Piezo + PRF (G3)	10	0	39	6.50	9.30	11.50			47.0 (G1–G4)	0.853
Traditional (G4)	10	2	53	26.01	26.16	19.45			98.0 (G2–G3)	0.631
									19.50 (G2–G4)	0.019*
									23.5 (G3–G4)	0.043*

Abbreviations: *Anal* Analgesics, *Min* minimum, *Max* maximum, *SD* standart deviation, *χ* ^*2*^ Kruskal Wallis test result; *p ≤ 0.05, significance **Mann–Whitney *U*-test (Bonferroni-correction) results between groups

**Table 4:** Average of trismus at 2nd, 3rd and 7th days after surgery, (%)

Groups (G)	Trismus (n)	2nd postoperative day (mm)	3rd postoperative day	7th postoperative day
		Mean ± SD	Mean ± SD	Mean ± SD
Traditional (G1)	10	20.90 ± 17.83	16.21 ± 16.30	6.75 ± 11.84
Traditional+ PRF (G2)	10	8.70 ± 10.50	7.00 ± 9.40	2.00 ± 3.52
Piezo + PRF (G3)	10	7.60 ± 6.85	5.30 ± 7.55	0.80 ± 1.61
Traditional (G4)	10	19.10 ± 19.45	15.60 ± 15.45	8.70 ± 13.15

The differences in trismus were not statistically significant between groups on the second (*χ* ^2^ = 5.355, p = 0.148), third (F = 1.985, p = 0.134) and seventh (*χ* ^2^ = 2.411, p = 0.492) postoperative days. Abbreviation: SD, Standart deviation; F, One-Way ANOVA test result; *χ* ^2^, Kruskal Wallis test result

### Swelling

On postoperative days 1, 3, and 7, there were no significant group differences in swelling (*p* > 0.05). Only difference was on the second day between the groups 1–4 (p = 0.018) and 2–4 (0.006), (Table 5).

**Table 5:** Average of swelling at 1st, 2nd, 3rd and 7th days, (%)

Groups (G)	Swelling (n)	1st postoperative day (mm)	2nd postoperative day (mm)	3rd postoperative day	7th postoperative day
		Mean ± SD	Mean ± SD	Mean ± SD	Mean ± SD
Traditional (G1)	10	2.20 ± 1.80	1.66 ± 1.71	1.15 ± 1.33	0.00 ± 0.00
Traditional + PRF (G2)	10	2.10 ± 1.37	1.40 ± 0.96	0.80 ± 0.63	0.00 ± 0.00
Piezo + PRF (G3)	10	3.00 ± 1.24	2.30 ± 1.16	1.40 ± 1.00	0.00 ± 0.00
Traditional (G4)	10	3.70 ± 1.63	3.00 ± 0.81	2.00 ± 0.94	0.00 ± 0.00

The differences in facial swelling were not statistically significant between groups on the first (F = 2.397, p = 0.084), third (F = 2.421, p = 0.082) and seventh postoperative days. Only difference was on the second day (F = 3.478, p = 0.026) between the groups 1–4 (p = 0.018), 2–4 (p = 0.006)

## Discussion

This study compared the surgical outcomes (pain, number of analgesics taken, swelling, trismus) after extraction of mandibular third molars using PRF and piezosurgery combined with PRF compared to standard rotating handpiece.

In the present study, the combined use of piezosurgery and PRF significantly decreased the number of analgesics taken. However, when PRF was not combined with piezosurgery, there were no statistically differences in the number of analgesics taken compared to groups that used traditional handpieces (the mean values: 9.4, 5.6, 4.3, 9.5, from group 1–4, respectively). In general, piezosurgery decreased the number of analgesics taken, which was in accordance with Barone et al. [8] and Goyal et al. [27]. In addition, the mean value of total VAS scores were so close in groups 2 and 3 (25.00, 24.45, respectively). According to this results, in impacted third molar surgery, the combined use of PRF and piezosurgery reduced pain more than the use of PRF after traditional surgery.

VAS has been proven to be a reliable and sensitive method for recording pain after oral surgery procedures which is straightforward to apply and widely used [1, 5, 6, 8–13, 15, 16, 18, 19, 25–27]. There are many authors, indicated in their studies that, using PRF is effective in reducing pain, in their studies, patients were recorded to either have no severe pain, significantly less pain or even no pain [17, 19, 21, 28–30]. Although further studies would be needed to deepen the knowledge of this biomaterial to determinate by which mechanism can it reduce pain.

In the literature there are few studies which show the effect of PRF for the control of pain, swelling, and trismus following the extraction of mandibular third molars. Kim et al. [31] reported that the use of PRF had no effect on pain following the surgical removal of impacted mandibular third molars and Singh et al. [19] also reported that PRF had no effect on pain following removal of mandibular third molars (no impaction), similar to that observed by Kim et al. [31]. However, in the present study, PRF significantly reduced pain. In the studies by Kim et al. and Singh et al., all of the patients underwent bilateral removal of impacted third molars during a single appointment. In our study, a minimum of 21 days separated the two operations in each patient for the return of parameters to preop-baseline prior to commencing second operation. In addition, the selection of processes of which technique to use first on each patient were randomly selected. The patients of Kim et al. and Singh et al. might not have been able to distinguish the level of pain on each side. This could be because the patients in both of these studies underwent bilateral removal of the third molars during a single appointment. However, the extent of swelling observed in our study was similar to that observed by Kim et al. [31].

The extent of trismus was significantly less in the groups treated with PRF (%9.03) and piezosurgery combined with PRF (%9.3), compared to the traditional handpieces used in group 1 (%25.61) and group 4 (%26.16) at the first day visits for postoperative interincisal distance, which was used for the evaluation of trismus.

Various methods have been used to measure facial swelling [25]. Our method was modification of tape measuring method of Gabka and Matsamura which was described by Ustun et al. [25]. Although it is not as accurate as computed tomography (CT) scan or magnetic resonance imaging (MRI) and does not make precise measurements of facial soft tissue volume, it is a noninvasive, simple, cost-effective and timesaving method, which provides us with numeric data for determination of soft tissue contour changes. Our study indicated no significant differences on swelling among the techniques used.

The degree of surgical difficulty was evaluated based on anatomic factors (depth of inclusion and ramus relationship) and the position of the third molar as assessed on radiographic examination. This has been reflected in the classification of Pell and Gregory [22]. In our study all the included mandibular third molars were of moderate difficulty (class I, level C). This condition was considered criteria for inclusion to reduce the risk of confounding factors and to obtain adequate homogeneity between the 2 groups.

Not only anatomical factors but bone cutting, sectioning of tooth, flap design, use of rotary instrument, time taken for surgical procedure and factors associated with operator are also accountable for incidence of complications. In the present study, we excluded the case if tooth needed sectioning during the surgery, flap design was triangular in shape in all extractions, there was no statistical difference between surgery durations, all of the examinations and extractions were done by same surgeon to optimize the homogeneity between the groups. In addition operation side and age distributions between the groups were homogeneous.

## Conclusions

The use of PRF, and PRF combined with piezosurgery, significantly reduced pain. In addition, PRF in combination with piezosurgery significantly decreased the number of analgesics taken. Both operations also significantly decreased trismus 24 h after the surgery. As a result of this study, PRF and combination use of PRF and piezosurgery have positive effects in reducing postoperative outcomes after impacted third molar surgery.
